# Mental Health Outcomes Among Homeschooled and Traditionally Schooled Adolescents in Egypt (Ages 12–18): A Cross-Sectional Study

**DOI:** 10.7759/cureus.95444

**Published:** 2025-10-26

**Authors:** Abdulkarim Hasan, Ahmed Ismael, Menna Sameh Zayed, Mahmoud M Abdelwahed, Ahmed M Abdelwahed, Mariam Tarek A Abubakr, Mohamed Fouad M Ragab, Mohammad M Badran

**Affiliations:** 1 Pathology, Faculty of Medicine, Al-Azhar University, Cairo, EGY; 2 Psychiatry, Mental Health Hospital - Al-Baha, Ministry of Health, Baljurashi, SAU; 3 Medicine and Surgery, Faculty of Medicine Cairo University, Cairo, EGY; 4 Medicine and Surgery, Faculty of Medicine, Galala University, Suez, EGY; 5 Medicine and Surgery, College of Medicine, Misr University for Science and Technology (MUST), Cairo, EGY; 6 Psychiatry, Yanbu General Hospital, Ministry of Health, Yanbu, SAU; 7 Psychiatry, Prince Mishari Bin Saud Hospital, Al-Baha Health Cluster, Baljurashi, SAU

**Keywords:** anxiety, dass-21, depression, homeschooling, mental health, stress

## Abstract

Background: Adolescence is a critical stage for the development of mental health, with depression, anxiety, and stress being among the most common concerns. Educational environments are key determinants of adolescent well-being, yet limited evidence exists on how homeschooling compares with traditional schooling in low- and middle-income countries such as Egypt.

Objective: This study aimed to compare depression, anxiety, and stress levels among homeschooled and traditionally schooled Egyptian adolescents aged 12-18 years.

Methods: A cross-sectional, questionnaire-based survey was conducted with 126 adolescents (20 homeschooled, 106 traditionally schooled). Participants completed the Depression, Anxiety and Stress Scale - 21 Items (DASS-21) in validated English and Arabic versions. Data were analyzed, and independent samples t-tests and chi-square tests were used to compare mean scores and categorical severity levels across groups.

Results: Depression of varying severity was identified in 32.5% of all studied students, anxiety in 45.2%, and stress in 11.9%. Female adolescents reported significantly higher depression, anxiety, and stress scores than males (p < 0.01). However, no significant differences were found between homeschooling and traditional schooling groups in mean scores or severity classifications for depression (p = 0.326), anxiety (p = 0.069), or stress (p = 0.052).

Conclusion: Female adolescents exhibited significantly higher levels of depression, anxiety, and stress compared to males, while the educational setting was not found to be significantly associated with mental health outcomes. Future studies with larger and more balanced samples, and preferably longitudinal designs, are recommended to further explore causal relationships and enhance generalizability across diverse educational contexts in Egypt and the wider Arab region.

## Introduction

Adolescence represents a critical developmental stage characterized by the formation of social, emotional, and behavioral patterns that underpin long-term mental health. During this period, the establishment of healthy sleep routines, regular physical activity, adaptive coping and problem-solving skills, positive interpersonal relationships, and effective emotion regulation is crucial. Globally, an estimated 17.5-20% of adolescents experience a mental health disorder, contributing substantially to the global burden of disease in this age group. Supportive environments within the home, school, and community contexts play a critical role in fostering these protective factors and promoting psychological well-being [1,2].

The mental health of adolescents is determined by multiple interacting factors. Key determinants include personal factors, such as biological and psychological characteristics, as well as environmental influences, including family, school, and peer relationships. This conceptual approach aligns with the biopsychosocial model, which emphasizes the dynamic interaction among biological, psychological, and social domains in determining mental health outcomes. Together, these factors play a central role in shaping adolescents’ mental well-being [2].

Between 2009 and 2019, the proportion of high school students reporting feelings of sadness and hopelessness increased markedly, rising from 26.1% to 36.7%. Over the same period, rates of suicide planning and suicide attempts also showed an upward trend [3].

In the United States, the prevalence of major depressive episodes among adolescents aged 12-17 years increased from 8.1% in 2009 to 15.8% in 2019 [4]. In China, a study of 4,100 students aged 11-16 years reported a depression prevalence of 34.0% [5], while a meta-analysis of 144,000 adolescents estimated the rate at 24.3% [6].

Access to accurate data on adolescent depression in different conditions is critical for developing and implementing effective prevention and intervention strategies.

Anxiety has a significant influence on students’ academic outcomes, as it reduces learning capacity and interferes with optimal performance [7].

Anxiety among students has detrimental effects on both mental and physical health, which in turn negatively impacts their academic achievement and future career development.

Stress is a common outcome of the pressures students face in today’s highly competitive academic environment. Multiple factors, including social media use, academic demands, and family or social relationships, strongly influence their performance. According to Aafreen et al. [8], students are consistently exposed to pressures from various sources throughout their academic life, which significantly contributes to elevated stress levels.

Recent discussions have increasingly highlighted the growing crisis within the education system, with researchers identifying several mechanisms through which traditional schooling may contribute to adolescent psychological distress. These include academic and examination pressure, competitive classroom environments, strained teacher-student relationships, and exposure to bullying or peer victimization. Such stressors have been associated with elevated levels of anxiety, depression, and emotional exhaustion among students. One approach to examining these issues is to compare the prevalence of emotional and behavioral problems among adolescents in traditional schooling and those receiving home education [9].

In Egypt, traditional schooling remains the predominant educational pathway, characterized by centralized curricula, limited flexibility, and examination-driven instruction, which may influence students’ mental well-being and access to physical activity in different ways depending on gender, socioeconomic status, and urban-rural disparities [10]. In recent years, however, alternative educational models have gained attention, particularly homeschooling, which is gradually being adopted by some Egyptian families - especially those residing abroad, where homeschooling is more socially and institutionally accepted [11]. In the Egyptian context, homeschooling generally operates outside the formal education framework, as families often register their children in national or international programs to maintain eligibility for recognized certification while conducting most of the educational process at home. Most homeschooled Egyptian students reside abroad and follow the Egyptian national curriculum, sitting for national examinations alongside their traditionally schooled peers. This evolving pattern reflects the growing, though still limited, adoption of homeschooling among Egyptian and expatriate families. The homeschooling population in Arab countries presents a unique challenge for researchers, as the existing body of literature remains scarce [12]. Although the number of homeschoolers continues to increase globally, including in Egypt, no official statistics are currently available on the size of this population. According to national data, approximately 7.3% of individuals aged four years and above have dropped out of formal education, which may reflect broader educational transitions and alternative learning practices [12].

In this study, we aim to compare mental health outcomes, specifically depression, anxiety, and stress, between homeschooled and traditionally schooled Egyptian adolescents aged 12-18 years. Using the Depression, Anxiety and Stress Scale - 21 Item (DASS-21) scale, we sought to assess whether differences in educational setting are associated with variations in psychological distress. By exploring these associations, our study contributes to the limited evidence on homeschooling in low- and middle-income countries and provides insights that may inform educational policies and mental health interventions tailored to adolescents in Egypt.

## Materials and methods

Study design

This study employed a cross-sectional, questionnaire-based survey design to compare mental health outcomes between homeschooled and traditionally schooled adolescents aged 12-18 years in Egypt. This age range was selected to correspond to the typical span of middle-to-secondary school education in the Egyptian system, ensuring that all participants were comparable in terms of educational stage and developmental context.

Sample size calculation

Sample size was estimated using Cochran’s formula for population proportions, assuming a moderate effect size, a 95% confidence level, and an 8% margin of error. This yielded a minimum required sample of 120 participants. A total of 126 adolescents were recruited, ensuring adequate statistical power.

Participants and sampling

Participants (n = 126) were recruited through convenience sampling, using social media networks for homeschooling communities and direct outreach to conventional schools. Eligible participants were Egyptian adolescents aged 12-18 years.

The inclusion criteria were as follows: (1) adolescents aged 12-18 years; (2) exclusive enrollment in either homeschooling or traditional schooling in Egypt; and (3) provision of both signed parental consent and adolescent assent. The exclusion criteria included the following: individuals outside the age range; enrollment in hybrid or alternative educational systems; non-Egyptian nationality; and responses that were incomplete, duplicated, or lacking valid consent or assent.

Data collection tools

Data were collected electronically using Google Forms to record demographic information and responses to the DASS-21. Both English and Arabic versions of the DASS-21 were provided, with the Arabic version previously validated by Alalalmeh et al. [13].

The DASS-21 consists of 21 items distributed equally across three subscales assessing depression, anxiety, and stress (seven items each). Responses are rated on a four-point Likert scale (0-3), reflecting the degree to which each statement applied during the past week. Scores for each subscale were doubled in accordance with Lovibond’s guidelines to align with the DASS severity classifications, ranging from normal to extremely severe [13,14] (Table 1).

**Table 1:** Severity Classifications (DASS-21 after ×2 adjustment) DASS-21: Depression, Anxiety and Stress Scale - 21 Items

Subscale	Normal	Mild	Moderate	Severe	Very Severe
Depression	0–9	10–13	14–20	21–27	28+
Anxiety	0–7	8–9	10–14	15–19	20+
Stress	0–14	15–18	19–25	26–33	34+

Statistical analysis

Data were analyzed using Statistical Product and Service Solutions (SPSS, version 22; IBM SPSS Statistics for Windows, Armonk, NY). Reliability of the DASS-21 scales was assessed using Cronbach’s alpha. Descriptive statistics were calculated for demographic variables and DASS-21 scores. Spearman’s rho was used to examine correlations among the DASS subscales. Independent samples t-tests compared mean depression, anxiety, and stress scores by gender, schooling type, and age group, while chi-square tests assessed categorical severity distributions. Statistical significance was set at p < 0.05.

## Results

A total of 126 adolescents participated in the study, distributed between homeschooled (n = 20) and traditionally schooled (n = 106) groups. The sample included 60 males (47.6%) and 66 females (52.4%). The majority of participants were in the older age group (15-18 years, 88.1%), while a smaller proportion were aged 12-15 years (11.9%).

Depression of varying severity was identified in 41 of 126 students (32.5%), anxiety in 57 students (45.2%), and stress in 15 students (11.9%)

Reliability of measures

Cronbach’s alpha was used to assess the overall reliability of the DASS (21 items across the three subscales: depression, anxiety, and stress). Prior to the analysis, items with item-total correlation coefficients below 0.60 were excluded at a 95% confidence level. The reliability analysis yielded a Cronbach’s alpha of 0.938 for the total scale (Table 2), indicating a very high level of internal consistency.

**Table 2:** Reliability statistics

Cronbach's Alpha	No. of Items	Subscale
0.813	7	Depression
0.841	7	Anxiety
0.846	7	Stress
0.938	21	Total

Correlations between the DASS-21 domains

Table 3 presents the results obtained using Pearson’s method to calculate the correlation coefficients among the three DASS constructs (depression, anxiety, and stress). The findings indicate strong positive correlations, with coefficients ranging from 0.792 to 0.844, all significant at p < 0.001. These results demonstrate a high degree of consistency and interrelatedness among the DASS measures of mental health.

**Table 3:** Correlations between the DASS-21 domains among the studied adolescents **: Correlation is significant at the 0.01 level (2-tailed) DASS21: Depression, Anxiety and Stress Scale - 21 Items

Subscale	Depression	Anxiety	Stress
Depression	1	0.792^**^	0.844^**^
Anxiety	0.792^**^	1	0.793^**^
Stress	0.844^**^	0.793^**^	1

Gender differences

Significant gender differences were observed across all three subscales. Female adolescents reported higher levels of psychological distress compared to males. Specifically, mean depression scores were significantly higher in females (M = 9.74, SD = 4.98) than in males (M = 6.10, SD = 4.08; p < 0.001). Similar trends were noted for anxiety (females: M = 8.94, SD = 5.52; males: M = 6.27, SD = 4.44; p = 0.004) and stress (females: M = 8.33, SD = 5.27; males: M = 5.67, SD = 5.15; p = 0.005). These findings suggest that female adolescents experience greater vulnerability to symptoms of depression, anxiety, and stress.

Education type

Comparisons between homeschooling and traditional schooling groups revealed no statistically significant differences in mean depression (p = 0.326), anxiety (p = 0.069), or stress (p = 0.052) scores. While mean values for homeschooled adolescents tended to be slightly higher in anxiety and stress, these differences did not reach statistical significance, indicating broadly comparable levels of psychological distress across schooling types (Table 4).

**Table 4:** Descriptive statistics of all sample and correlations between all variables

Subscale	Characteristics		N	%	Mean Score	Std. Deviation	P value	t
Depression	Gender	Male	60	47.6	6.10~6	4.078	0.000	-4.46
		Female	66	52.4	9.74~10	4.984		
	School	Traditional	106	84.1	7.82~8	4.792	0.326	-0.98
		Home	20	15.9	9.00	5.516		
	Grade	Middle School	15	11.9	5.73~6	5.535	0.056	-1.93
		Secondary School	111	88.1	8.32~8	4.763		
Anxiety	Gender	Male	60	47.6	6.27~6	4.441	0.004	-2.97
		Female	66	52.4	8.94~9	5.524		
	School	Traditional	106	84.1	7.30~7	5.020	0.069	-1.83
		Home	20	15.9	9.60~10	5.789		
	Grade	Middle School	15	11.9	5.80~6	6.236	0.139	-1.49
		Secondary School	111	88.1	7.92~8	5.017		
Stress	Gender	Male	60	47.6	5.67~6	5.148	0.005	-2.86
		Female	66	52.4	8.33~8	5.269		
	School	Traditional	106	84.1	6.66~7	5.121	0.052	-1.96
		Home	20	15.9	9.20~9	6.195		
	Grade	Middle School	15	11.9	4.80~5	5.532	0.081	-1.75
		Secondary School	111	88.1	7.37~7	5.288		

School type (classification of school)

No significant differences were found between the two groups (middle vs secondary) in depression (p = 0.056), anxiety (p = 0.139), or stress (p = 0.081). Although older adolescents tended to report marginally higher mean scores, the differences were not statistically meaningful.

Symptom severity distributions

Analysis of categorical severity classifications revealed similar patterns between schooling groups. For depression, the majority of both homeschooled and traditionally schooled adolescents fell into the normal to mild range, with very few cases reaching the severe category. Anxiety severity levels showed greater variability, with homeschooled adolescents having a slightly higher proportion in the moderate-to-severe categories compared to their traditionally schooled peers; however, chi-square analysis confirmed these differences were not significant (p = 0.624). Stress levels were predominantly normal in both groups, with fewer than 20% classified as mild or moderate, and no cases in the severe or very severe categories (Table 5).

**Table 5:** A comparison between traditional and home schooling according to DASS DASS: Depression, Anxiety and Stress Scale

DASS Symptoms		School				P value	Chi-square
		Traditional		Home			
		N	%	N	%		
Depression	Normal	72	67.92	13	65	0.612	1.81
	Mild	18	16.98	3	15		
	Moderate	15	14.15	3	15		
	Severe	1	0.94	1	5		
Anxiety	Normal	61	57.55	8	40	0.624	2.61
	Mild	14	13.21	3	15		
	Moderate	21	19.81	6	30		
	Severe	8	7.55	2	10		
	Very Severe	2	1.89	1	5		
Stress	Normal	95	89.62	16	80	0.051	5.96
	Mild	10	9.43	2	10		
	Moderate	1	0.94	2	10		

**Figure 1:**
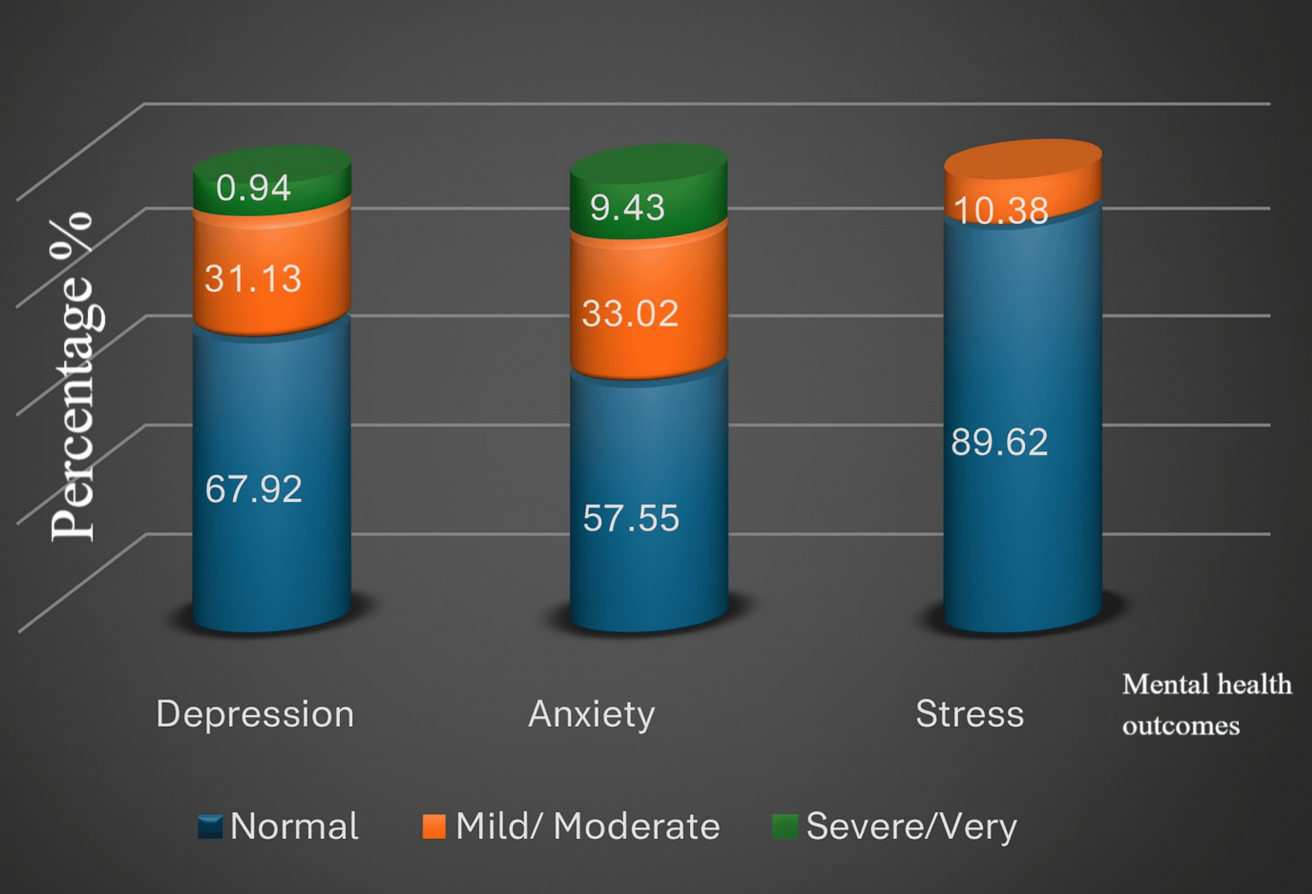
Stacked column chart illustrating the distribution of depression, anxiety, and stress levels among participants in the traditional schooling group, based on the DASS-21 DASS-21: Depression, Anxiety and Stress Scale - 21 Items

**Figure 2:**
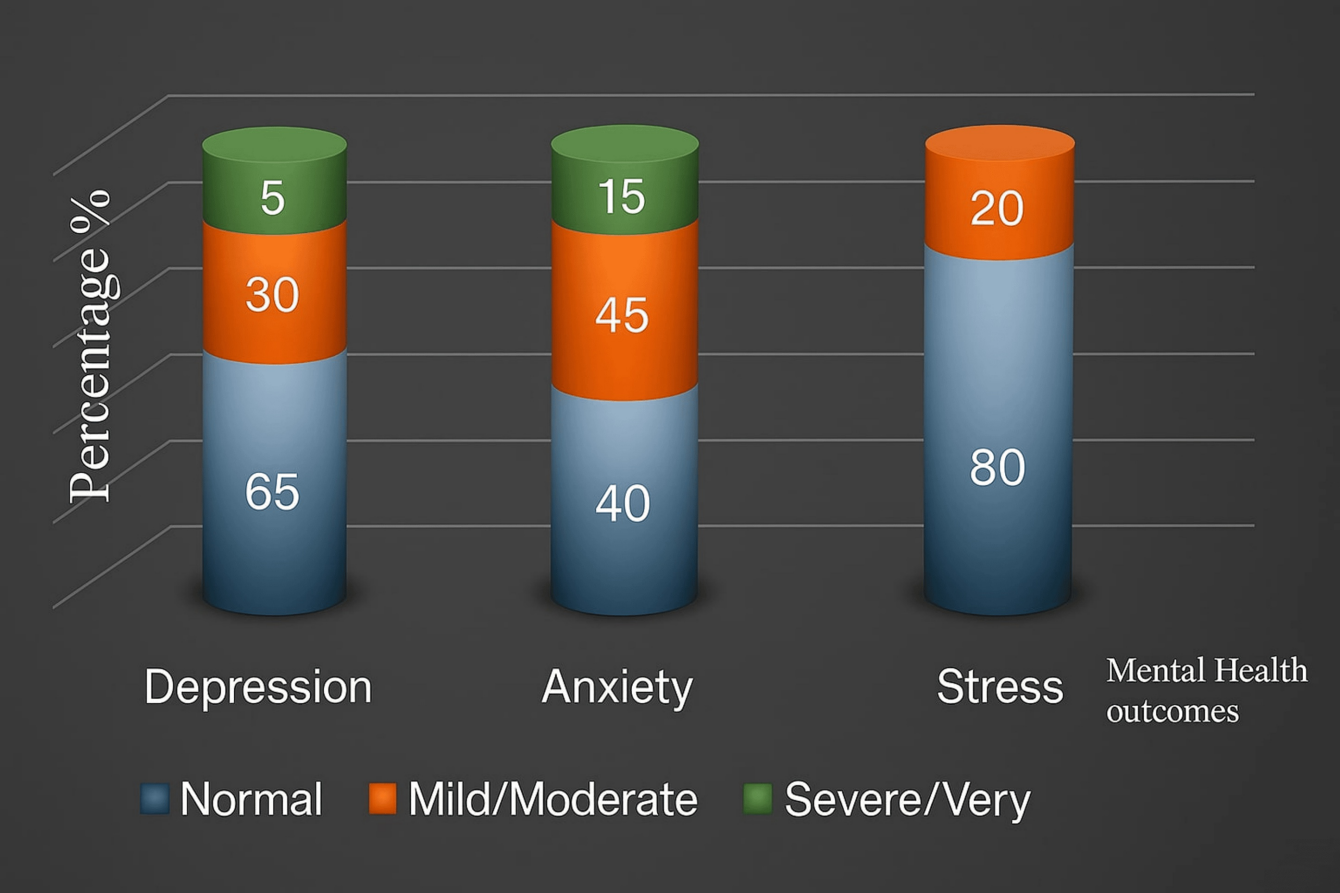
Stacked column chart illustrating the distribution of depression, anxiety, and stress levels among participants in the home-schooling group, based on the DASS-21 DASS-21: Depression, Anxiety and Stress Scale - 21 Items

To aid interpretation, the diseased categories were merged in the graphical representation, with mild and moderate combined into one group and severe and very severe combined into another group, as presented in Figures 1-2.

## Discussion

Mental health outcomes have been shown to be closely associated with individual characteristics and personality traits. Self-esteem and self-efficacy, which reflect cognitive evaluations of self-worth and perceived capability, are particularly influential [2,15]. Within the framework of social cognitive theory, perceiving oneself as unable to act effectively can generate anxiety, feelings of futility, and depressive states [16]. Several previous studies on adolescents support this, revealing associations between low self-efficacy, depressive symptoms, and anxiety disorders [17]. Moreover, cognitive factors demonstrate a bidirectional relationship with mental health, functioning both as risk factors for and consequences of psychological problems. Research has highlighted reciprocal links between self-esteem and outcomes, such as depression, anxiety, and overall well-being in adolescent populations [18]. Additionally, maladaptive perfectionism has been identified as a personality trait that heightens vulnerability to depression, anxiety, and poorer mental health among teenagers [19].

Education has important protective effects on mental health, with stronger impacts observed among women and rural populations. These benefits are well documented within formal educational settings, where structured environments, peer interaction, and institutional support contribute to psychological resilience. In homeschooling contexts, the nature of these protective effects may vary depending on parental involvement, social engagement opportunities, and access to learning resources. These effects appear to be mediated by improvements in physical health, health knowledge, and empowerment. Compared with short-term interventions, such as anti-poverty programs, expansion of education provides a more sustainable, large-scale, and cost-effective means of improving mental health [20,21]. Education can therefore be considered an effective policy tool with additional benefits beyond academic outcomes, highlighting the need for further research in developing countries on its broader influence on adolescent mental health [22].

Our results for Egyptian students show that depression was identified in 41 of 126 students (32.5%), anxiety in 57 students (45.2%), and stress in 15 students (11.9%). This result is consistent with the findings of Fawzy et al. in 2017, who studied the undergraduate Egyptian students and found that nearly one-third of undergraduates met the diagnostic criteria for moderate depression, emphasizing the significant role of developmental stage and academic stressors in shaping mental health outcomes in this population [23].

However, Essawy et al.'s study, assessing childhood depression among students aged 12-16 years, found that 10.5% of the sample (n = 739) had depressive symptoms [24]. Among those identified with symptoms, the severity was distributed as 40% mild, 24% moderate, and 36% severe depression, based on the Childhood Depressive Inventory (CDI).

Young women generally report poorer mental health outcomes compared to their male peers in Egypt, and lower academic performance was linked to heightened mental health difficulties, according to Liu et al. [25].

Significant gender differences were observed in our study across all three subscales. Female adolescents reported higher levels of psychological distress compared to males. Specifically, mean depression scores were significantly higher in females than in males.

The higher mean depression scores observed in females may be influenced by a combination of biological sensitivity, psychological coping styles, and certain social factors, which could contribute to greater vulnerability among young women compared to men [26].

Anxiety negatively affects students’ academic achievement by reducing their learning capacity and limiting optimal performance. Broadly, five key factors - personal, familial, institutional, social, and political - can contribute to the development of severe anxiety disorders in students [27]. Anxiety among students has been shown to adversely affect both their mental and physical health, with potential long-term consequences for their academic and career trajectories.

Our results indicate that there is no significant difference between homeschooled and traditionally schooled students in terms of anxiety scores or academic grades. However, a significant gender difference was observed, with female students reporting higher anxiety scores than their male counterparts.

Several previous studies on Egyptian adolescents have highlighted anxiety as a prevalent issue, particularly among female students and those dissatisfied with their academic performance, with strong associations to diminished quality of life [25,26,28].

Gender differences in psychological distress have long been a subject of research. Epidemiological studies generally suggest that women are more prone to mental health problems than men, for two main reasons. First, biological factors, including genetic susceptibility, hormonal influences, and cortisol levels, manifest emotionally and behaviorally. For example, men and women show different stress responses due to variations in sensitivity to stressful events. Women tend to be more vulnerable to stress and pain, which may contribute to higher levels of sadness and anxiety [29].

Second, socially prescribed gender roles shape patterns of internalizing and externalizing problems. Evidence indicates that women are more likely to experience internalizing disorders such as depression and anxiety, whereas men are more often associated with externalizing disorders [30].

While gender differences in mental health have been well-documented in the general population, the literature review indicated that evidence is still very limited among homeschooled and traditionally schooled students. Previous studies have reported psychological effects of stay-at-home measures; therefore, it can be expected that prolonged confinement at home, without attending school or engaging in outside activities, may increase feelings of loneliness, anxiety, and stress [31,32]. In the present study, however, no significant differences were found between homeschooled and traditionally schooled students in relation to depression, stress, or anxiety. Nevertheless, homeschooling is closely associated with the ideology of intensive mothering, a dominant parenting style in the United States that has been linked to greater maternal distress [33].

The absence of significant differences between the homeschooling and traditional schooling groups in the present study may be attributable to several factors, including the shared academic structure. Most homeschooled students follow the same national curriculum and undertake formal examinations, which may produce similar educational pressures and learning experiences across both settings.

This study contributes to the limited evidence on homeschooling in low- and middle-income countries and underscores the need for comprehensive, multi-country research that considers cultural, familial, and socioeconomic factors. Such investigations are essential to inform policymakers and educators in developing evidence-based strategies to promote adolescent well-being across different educational settings. Future studies should also examine how homeschooling, particularly within the context of intensive mothering, may affect maternal mental health and overall family dynamics.

Limitations of the study include the following: (a) the relatively small sample size and focus on Egyptian adolescents, which may restrict the generalizability of the findings to wider populations; (b) the unequal group sizes, as homeschooling is relatively uncommon in Egypt compared to traditional schooling, which limited the number of homeschooled participants and may have reduced the statistical power; (c) the use of social media platforms for recruitment, which may have introduced selection bias; (d) the cross-sectional study design, which limits causal interpretation; and (e) reliance on self-reported questionnaires, which may introduce reporting bias as participants could under- or overestimate their symptoms.

## Conclusions

This study compared depression, anxiety, and stress levels between homeschooled and traditionally schooled adolescents in Egypt, identifying depression in 32.5%, anxiety in 45.2%, and stress in 11.9% of participants. Although no statistically significant differences were found between schooling types, female students consistently reported higher psychological distress. These findings highlight gender as a key determinant of adolescent mental health, while the absence of significant group differences should be interpreted cautiously given the small sample size. Further studies with larger, more balanced samples are recommended to validate these findings and explore potential contextual and causal factors.
